# Editorial for the Special Issue: “Current and Novel Antimicrobial Strategies for Bacterial and Fungal Infections by Resistant Organisms”

**DOI:** 10.3390/antibiotics11040426

**Published:** 2022-03-23

**Authors:** Alberto Enrico Maraolo

**Affiliations:** First Division of Infectious Diseases, Cotugno Hospital, AORN Ospedali dei Colli, 80131 Naples, Italy; albertomaraolo@mail.com

The pandemic driven by the SARS-CoV-2 infection has compelled health services globally to direct all available human and economic resources toward fighting the novel coronavirus. Nevertheless, another pandemic looms over both high-income and low-income countries—the one represented by antimicrobial resistance (AMR).

A 2019 systematic analysis investigated the impact of AMR in 204 countries and territories, just before the global breakout of SARS-CoV-2. It estimated that nearly 5 million deaths were associated with bacterial AMR in 2019, of which a quarter (1.27 million) were directly attributable to bacterial AMR [1]. The highest death rate across all ages attributable to AMR itself was predicted to have occurred in Western sub-Saharan Africa [1].

Therefore, it is imperative to stay vigilant to resistant organisms, to prevent another severe crisis from affecting healthcare systems, which are already under strain due to the 2019 coronavirus (COVID-19) pandemic. Besides rigorous infection control policies at national and local levels, strategies to optimize antimicrobial weapons are necessary. For instance, there is a need for stewardship initiatives aimed at containing the misuse of carbapenems by correctly using alternative drugs when feasible [2], especially considering that carbapenem-resistant bacteria are among the major drivers of AMR-related deaths [1]. Other strategies may include the streamlined use of old and repurposed agents such as colistin and other drugs [3].

This Special Issue includes full research articles, reviews and brief reports addressing the issue of AMR from several perspectives; these include risk factors and molecular aspects, with a focus on therapeutics, and particularly on new drugs or novel approaches.

Regarding the first type of contribution, concerning the risk factors of AMR, Baraka and colleagues presented an interesting cross-sectional survey aimed at physicians, nurses and pharmacists from six tertiary hospitals in the Eastern province of Saudi Arabia [4]. Respondents acknowledged that improper antimicrobial use based on indication, duration and type were, unfortunately, common practice, and were identified as key factors contributing to AMR. According to the authors, the relevant knowledge gap must be filled in that area of Saudi Arabia to improve the appropriateness of antimicrobial use.

Silago and coworkers reported on the existence of multiple extended-spectrum beta-lactamase (ESBL) genes (*bla*_CTX-M_, *bla*_TEM_, and *bla*_SHV_) among *Enterobacterales* spp., isolated from multiple samples in a nosocomial setting in Tanzania [5]. Isolates harboring all three ESBL genes showed complex resistant patterns to antibiotics belonging to non-beta-lactam classes. Furthermore, the study found isolates of *Klebsiella pneumoniae* harboring *bla*_CTX-M_ and *bla*_TEM_ from the contaminated hands of healthcare workers, highlighting the potential role of personnel in the transmission of ESBL-producing pathogens.

Regarding therapeutic aspects, this Special Issues included papers specifically describing current and novel antimicrobial strategies.

Although not considered a resistant organism, *Clostridioides difficile* is regarded as a major and urgent antibiotic-resistant threat, along with carbapenem-resistant pathogens. Indeed, *C. difficile* is caused by the same factors that drive antibiotic resistance, namely antibiotic use responsible of dysbiosis and the spread of germs. Oral vancomycin is a long-standing option for the first-line treatment of *C. difficile* infection, as well as for severe and recurrent forms. Recently, oral vancomycin prophylaxis (OVP) has emerged as a preventative strategy against either primary or secondary (recurrent) *C. difficile* infection in subjects undergoing concurrent systemic antibiotic therapy (SAT). Maraolo and colleagues performed a comprehensive systematic review with meta-analysis to pool available evidence on the topic; they showed that OVP is a very promising approach [6]. Of course, there are many potential strategies to minimize the risk of primary *C. difficile* infection or recurrence, but oral vancomycin appears to be safe and inexpensive. At any rate, more data from randomized clinical trials are needed. In the meantime, OVP should be offered to selected persons, such as immunosuppressed patients undergoing SAT based on drugs associated with a high risk of *C. difficile* infection.

When it comes to novel therapeutic options for resistant bacteria, latest generation cephalosporins are among the main characters on the stage.

Giacobbe and collaborators illustrated the real-life use of ceftaroline in a tertiary hospital located in northern Italy [7]. Ceftaroline is a fifth-generation cephalosporin whose spectrum of activity includes methicillin-resistant *Staphylococcus aureus* (MRSA). This makes the agent a very appealing option against a vast array of infections, beyond the on-label indications represented by complicated infections of the skin and soft-tissue, as well as community-acquired pneumonia. The cohort consisted of 200 patients, from July 2019 to December 2020. Ceftaroline was used as empirical therapy in most cases (179/200), generally for suspected bacterial co-infection or superinfection in COVID-19 patients; only in 7% of patients was there an off-label use, usually for treating a bloodstream infection arising from MRSA.

Bavaro and colleagues focused instead on difficult-to-treat Gram-negative bacteria, specifically describing the effectiveness of cefiderocol-based combination regimens in 13 patients [8]. Cefiderocol is a novel siderophore cephalosporin that could be a game changer in the fight against carbapenem-resistant superbugs. Indeed, it was conceived to overcome the challenges linked to paramount carbapenem-resistance mechanisms exhibited by fermenters such as *Enterobacterales*, and by nonfermenters such as *Acinetobacter baumannii*, *Pseudomonas aeruginosa*, and *Stenotrophomonas maltophilia*. Cefiderocol is stable against hydrolysis by all carbapenemases, and its entry into bacterial cells is independent of porin channels and efflux pumps. Its place in therapy has not been established yet, nor has the best use for it as monotherapy or in combination with other agents. According to Bavaro and colleagues, cefiderocol was always associated with several companion drugs, and microbiological eradication was achieved in all 13 cases. Three patients died (outcome assessed at 30 days), but their deaths were attributed to causes other than the infections for which cefiderocol was utilized.

Strategies to properly counter severe infections may not rely only on antimicrobials. Recchia and coworkers illustrated a case report on a young male subject affected by Lemierre’s syndrome, whose aetiologic agent was *Fusobacterium necrophorum*—an anaerobic, non-spore-forming, non-motile, Gram-negative bacterium [9]. Physicians took an aggressive stance to counter the neuroendocrine dysregulation accompanying septic shock by exploiting the synergistic effect of norepinephrine, vasopressin and steroids. Of course, the patient was treated with proper antimicrobial treatment as well as a surgical source control, in accordance with two tenets of Lemierre’s syndrome therapy. This case report serves as reminder that serious infections, even when not caused by resistant organisms (although the rate of AMR among anaerobic bacteria is reportedly increasing worldwide), may need innovative therapeutic approaches to achieve good clinical outcomes. Ultimately, the aim of this Special Issue was to attract papers illustrating avant-garde approaches for fungal infections. To this end, Hefzy and colleagues reported on the antifungal inhibitory activity of bacteriocin-like inhibitory substances (BLISs); these were derived from clinical isolates of potentially probiotic lactobacilli and streptococci on *Candida* spp. (albicans and non-albicans) in women with vulvovaginitis [10]. The experiments were conducted in vitro and in vivo (using the *Galleria mellonella* larvae model), and yielded encouraging results that may be the basis for further research aimed at establishing BLISs as an alternative or adjunctive antifungal therapy for vulvovaginal candidiasis. Novel antimycotic options are required, since many pathogenic *Candida* species have developed resistance to the currently available antifungal agents.

In conclusion, this Special Issue collects multidisciplinary research articles that hopefully represent a fruitful contribution for stakeholders working in the field of AMR, either in bacterial or fungal-lactamases, or in related fields.
